# Coverage and Characterization of Food Delivery Services Through a Mobile Application in Small and Medium-Sized Cities in Brazil

**DOI:** 10.3390/ijerph22020293

**Published:** 2025-02-16

**Authors:** Renata Júlia da Costa, Paloma Aparecida Anastacio Barros, Juliana de Paula Matos, Paula Martins Horta

**Affiliations:** Department of Nutrition, Federal University of Minas Gerais, Av. Prof. Alfredo Balena, 190-Santa Efigênia, Belo Horizonte 30130-100, MG, Brazil; renatajulia@gmail.com (R.J.d.C.); ju.liana2011@yahoo.com.br (J.d.P.M.); paulamhorta@gmail.com (P.M.H.)

**Keywords:** food delivery apps, socioeconomic factors, digital food environment, ultra-processed foods

## Abstract

This is the first study to analyze the coverage and profile of establishments registered on a meal delivery application (MDA) in small and medium-sized cities in Brazil. The app serves 497 municipalities across 22 states, offering 28,325 establishments. App coverage was measured as the ratio of cities served to the total small and medium-sized cities in each Brazilian region. The establishments were categorized using keyword analysis into six groups: International Cuisine, Snacks, Bakery Products, Complete Meals and Side Dishes, Sweets, Ice Creams and Desserts, and Healthy Options. Greater app coverage was observed in the South (13.68%) and Southeast (13.63%) regions. In contrast, lower coverage was noted in the North (2.46%) and Northeast (2.30%) regions. The keyword category ‘Snacks’ was the most used across all Brazilian regions, while ‘Healthy Options’ was the least utilized. Positive correlations were identified between the number of establishments on the app and the Municipal Human Development Index (r = 0.30; *p* < 0.001), demographic density (r = 0.23; *p* < 0.001), and urban population (r = 0.55; *p* < 0.001). The use of keyword categories varied across municipalities based on their demographic and socioeconomic characteristics. Our findings reveal significant asymmetries in app coverage and the types of registered establishments, which may further exacerbate inequalities in food access.

## 1. Introduction

Meal delivery applications (MDAs) serve as platforms that connect consumers with food-selling establishments [1,2]. Aggregator platforms, such as iFood and Uber Eats, provide a wide range of establishment options [1,2,3]. These platforms allow consumers to explore menus and establishment details through search filters, which can include customer ratings, distance, price range, delivery time, or specific keywords [4]. Keywords enable establishments to categorize their offerings by cuisine type (e.g., “Brazilian”), food item (e.g., “burger”), meal (e.g., “lunch”), or appeals (e.g., “healthy”) [4,5].

The use of MDAs has been increasing both in Brazil and globally, primarily due to their convenience, time-saving benefits, and practicality [1,2]. The global online food delivery market was valued at USD 770 billion in 2022, with projected revenues of USD 1.45 trillion and an estimated 2.5 billion users by 2027 [6,7]. In Brazil, the sector’s revenue is expected to reach USD 4.6 billion by 2027 [8].

Frequent use of MDAs raises concerns from a health perspective, as recent evidence highlights the widespread availability of low-nutritional-quality foods on these platforms [3,4,5,9,10,11,12,13,14,15,16], alongside various marketing strategies that promote such foods [5,17,18,19]. These factors create an environment that may encourage unhealthy food choices, potentially contributing to adverse health outcomes, including excessive weight gain and the development of non-communicable chronic diseases (NCDs) [3,20].

MDAs are primarily prevalent in large urban areas, though they have also gained traction in smaller cities [1,2]. However, existing research predominantly focuses on market-leading aggregator platforms, such as Uber Eats [10,11,12,14,16,21], DoorDash [12], and Just Eat [15,21,22], operating in populous regions of developed countries [4,10,11,12,14,15]. In Latin America, a study examined establishments registered on an app across nine urban centers [5], while in Brazil, research has characterized the services of these platforms in major metropolitan areas like São Paulo [5], Belo Horizonte [17,19], and other state capitals [18]. As a result, there is limited knowledge about the scope of MDAs operating in small and medium-sized cities, as well as the characteristics of the establishments registered on these platforms.

This understanding is crucial, considering the large number of small and medium-sized cities in Brazil, which exhibit considerable cultural, economic, social, and urban diversity [23]. This study aimed to analyze the coverage and profile of establishments registered on an MDA in small and medium-sized cities in Brazil, according to the demographic and socioeconomic characteristics of these municipalities.

## 2. Materials and Methods

### 2.1. Study Design

This is a cross-sectional study. Small cities were defined as those with populations of up to 100,000 inhabitants, while medium-sized cities were those with populations ranging from 100,000 to 500,000 inhabitants [24,25].

### 2.2. Selection of the Application

Between 23 December 2022 and 1 January 2023, searches were conducted in the app stores of Android (Play Store) and iOS (App Store) using the keywords “meal delivery app” and “food delivery app.” The search yielded a total of 680 apps, with 355 in the Play Store and 325 in the App Store. After excluding 58 duplicate apps, 622 apps remained.

From this list, the following apps were excluded: (i) those not related to meal delivery (e.g., pharmacies, pet stores, games, or recipe apps); (ii) apps exclusive to a single network or brand (e.g., Burger King, KFC, McDonald’s); (iii) supermarket chain apps; (iv) apps not operating in Brazil (e.g., DoorDash, Uber Eats); and (v) apps unavailable for download at the time of data collection (e.g., Food Delivery). After applying these exclusions (n = 532), 90 apps remained.

**Figure 1:**
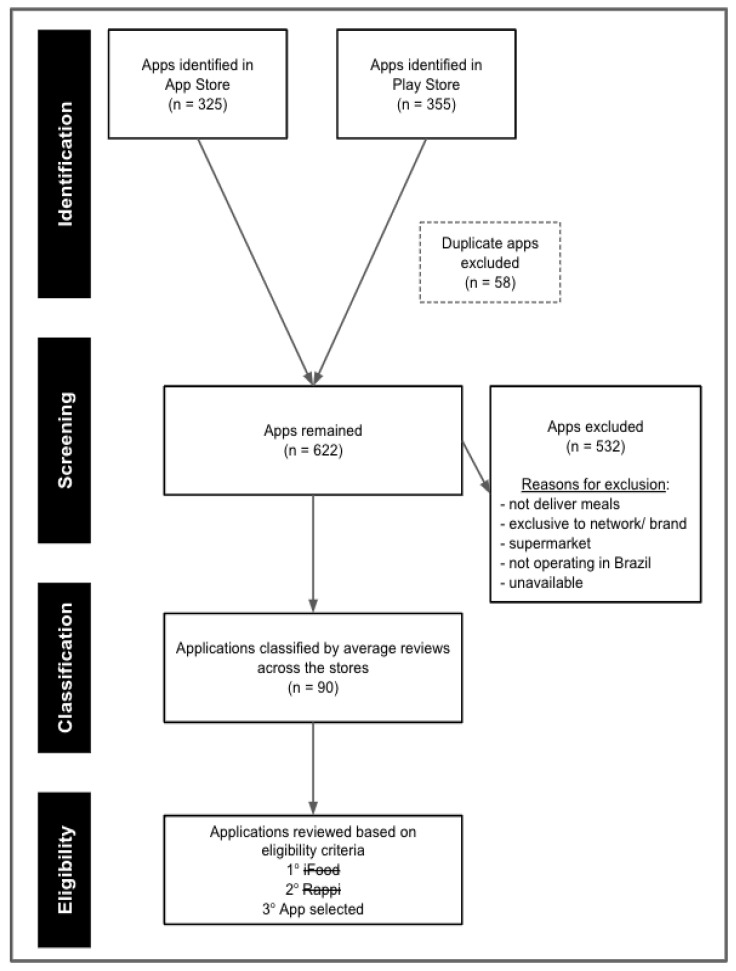
Flowchart depicting the application selection process.

For each app, the average number of user reviews was calculated as reported in the Play Store and App Store. The two main aggregation platforms in the country were averages of 9,450,000 (iFood) and 1,202,500 (Rappi) reviews, respectively. However, these apps primarily focus on large cities, which fall outside the scope of this study. The third app in the selection process, aiquefome, operates exclusively in small and medium-sized cities and has an average of 236,908 reviews. The selection process for this application is illustrated in Figure 1.

This company (aiquefome) is a Brazilian startup founded in 2007, with a focus on operations in cities with populations ranging from 15,000 to 300,000 inhabitants [26]. The platform primarily delivers ready-to-consume food, although it also offers e-commerce services for other products and establishments, including gas stations, grocery stores, pet shops, and smoke shops [27]. The company has over 5.8 million users, approximately 25,000 partner restaurants, and delivers over 33 million orders annually [26]. In 2021, it served 5.3 million customers across 700 Brazilian municipalities [27].

### 2.3. Selection and Characterization of Establishments Available on the App Using Keywords

A search was conducted on the company’s website to identify all Brazilian cities served at the time of the study (n = 497). Subsequently, all establishments registered on the app with available delivery in each city were retrieved. Data collection was carried out using the open-source Scrapy framework (https://scrapy.org/ accessed on 27 July 2023), which facilitates web crawling and data extraction through Python programming. This step was conducted on 27 July 2023, between 4:30 p.m. and 5:30 p.m. The search returned a total of 30,966 establishments, and for each, the following data were collected: establishment name, city, state, and keyword. The data for each city were organized and tabulated using Microsoft Excel version 16.80.

Following the methodology used by Poelman et al. and Matos et al., each establishment was identified based on the first keyword assigned to it [4,5]. This approach streamlines the analysis of a large number of establishments and minimizes the time required for menu evaluation [4,5]. Establishments were excluded based on the following criteria: (i) those whose keywords indicated they did not sell food (n = 750, e.g., pharmacies, pet shops); (ii) those that did not offer ready-to-eat meals (n = 371, e.g., butcher shops, supermarkets, hampers); (iii) those that sold alcoholic beverages (n = 1366, e.g., beer, drinks, convenience stores); or (iv) those that did not use identifying keywords (n = 154). Following these exclusions, 2641 establishments were removed, leaving 28,325 establishments for analysis.

A total of 122 different keywords were identified and categorized based on their similarities, following the methodology established by other studies [4,5]. The six keyword categories created were: ‘International Cuisine’; ‘Snacks’; ‘Bakery Products’; ‘Complete Meals and Side Dishes’; ‘Sweets’, ‘Ice Creams’, and ‘Desserts’; and ‘Healthy Options’ (Table 1). The participation of each keyword across the evaluated establishments is available in Supplementary Material S1.

### 2.4. Demographic and Socioeconomic Characterization of Cities Served by the App

For the demographic characterization of the cities, data on population density and the total population residing in urban areas were utilized. Both datasets were obtained from the 2010 Census conducted by the Brazilian Institute of Geography and Statistics (IBGE) [28]. Although the Brazilian demographic census was conducted in 2022, the data currently available for research still refer to the 2010 census.

For the socioeconomic analysis of the municipalities, the Municipal Human Development Index (HDI-M) was employed. This index incorporates indicators from three dimensions of human development: longevity, education, and income. The HDI-M ranges from 0 to 1, with values closer to 0 indicating lower levels of human development within the municipality [29]. The HDI-M corresponds to the year 2010, and data were sourced from the Human Development Atlas of the United Nations Development Program [30].

### 2.5. Analysis and Data Treatment

The coverage of the app was estimated by calculating the ratio between the number of cities served and the total number of small and medium-sized municipalities. The characterization of the establishments was carried out by estimating the frequency of keyword categories. Analysis was stratified by Brazilian regions: Central-West, North, Northeast, South, and Southeast. This classification is based on natural, social, cultural, and economic characteristics. The North and Northeast are the most economically disadvantaged regions. The Southeast holds significant economic importance and accounts for over 40% of the national population. The South, the smallest region, represents 14% of the population and is one of the wealthiest. The Center-West has experienced accelerated development since the establishment of Brasília as the capital in the 1960s, positioning it at an intermediate stage of development.

Additionally, differences in the participation of keyword categories among municipalities were assessed in relation to demographic and socioeconomic characteristics using Spearman’s correlation test, with a significance level set at 5%. This test was selected due to the non-normal distribution of the variables, as determined by the Shapiro-Wilk test (*p* > 0.05). All statistical analyses were conducted using Stata 14.0 software.

## 3. Results

The app serves 497 municipalities across 22 of the 26 states in Brazil, covering all five regions of the country. Its coverage in small and medium-sized municipalities nationwide is 8.99%, with the highest coverage observed in the South (13.68%) and Southeast (13.63%) regions. In contrast, the app reaches only 2.46% of the 447 small and medium-sized municipalities in the North region and 2.30% of the 1783 municipalities in the Northeast region (Table 2).

Regarding the keywords used by establishments, the ‘Snacks’ category was the most prevalent across all Brazilian regions, ranging from 44.07% in the Midwest to 51.88% in the South. The ‘Healthy options’ category was the least frequent, with 0.68% in the Midwest and 1.74% in the North. The ‘Complete meals and side dishes’ category was most used by establishments in the North region (32.84%) and least common in the Southeast region (23.03%). The ‘Sweets, ice creams, and desserts’ category was most prevalent in the Midwest (19.64%) and Southeast (19.24%) regions, and least in the North (11.48%). The ‘Bakery products’ category was most frequently used by establishments in the North (5.34%) and least in the South (2.62%). Finally, ‘International cuisine’ was most common in the South (5.23%) and least in the North (3.34%) (Table 3).

**Figure 2:**
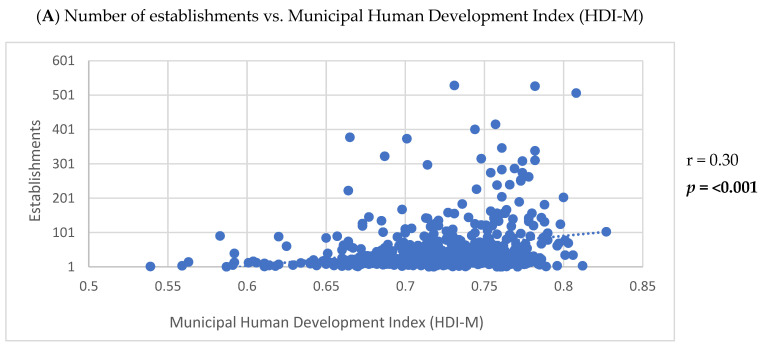

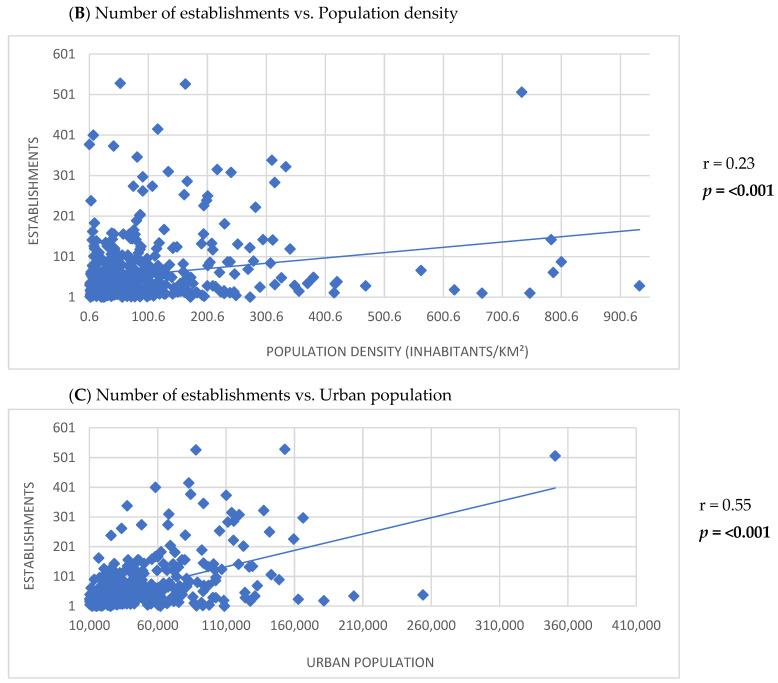
Correlation between the number of establishments listed in the meal delivery application and the demographic and socioeconomic characteristics of small and medium-sized cities.

**Figure 3:**
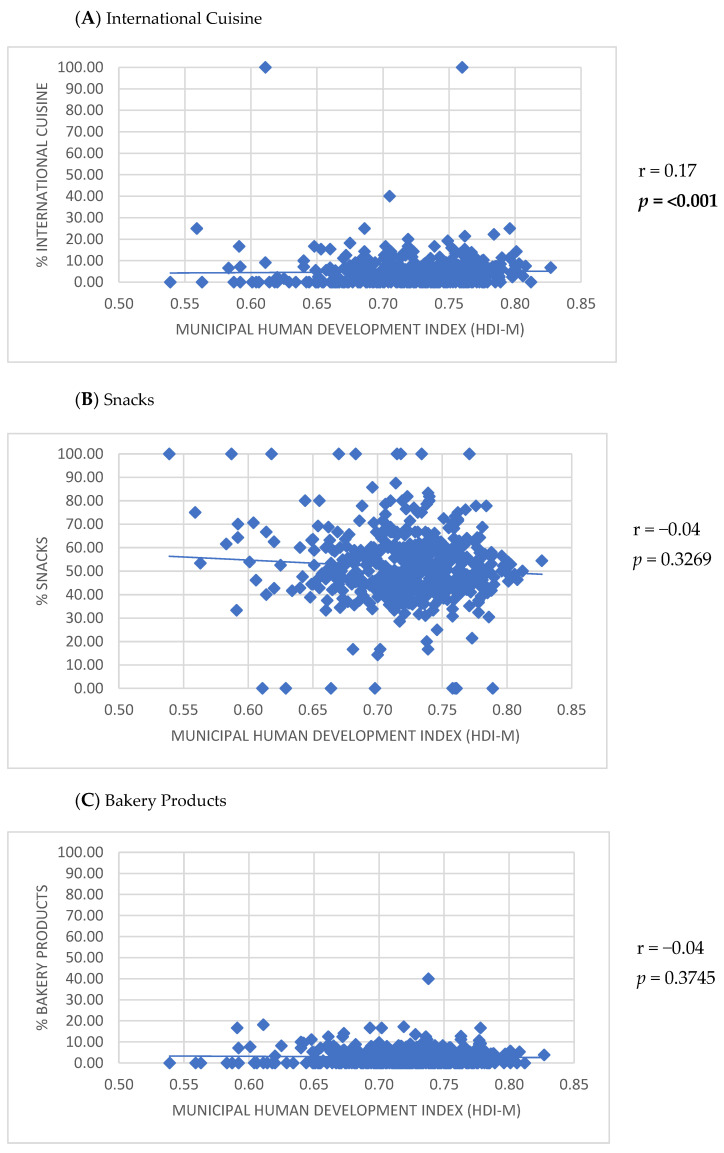

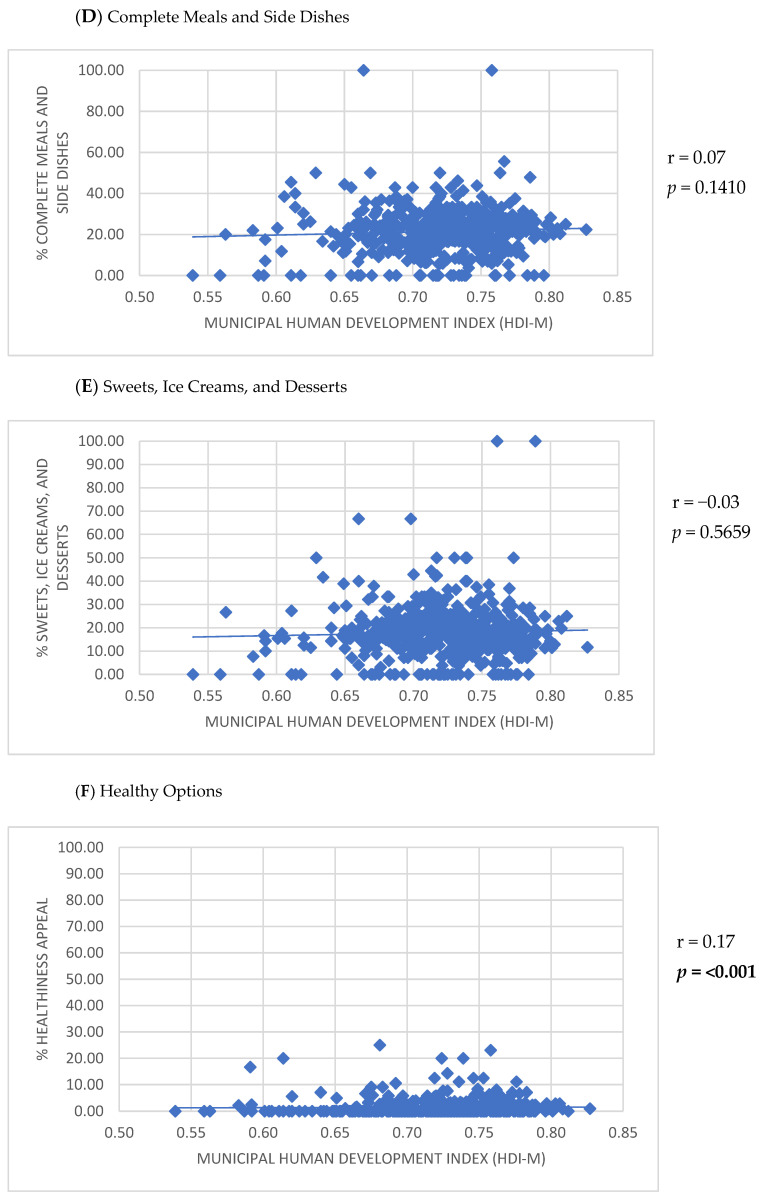
Correlation between the participation of keyword categories for establishments listed in the meal delivery application and the Municipal Human Development Index (HDI-M) of small and medium-sized cities.

**Figure 4:**
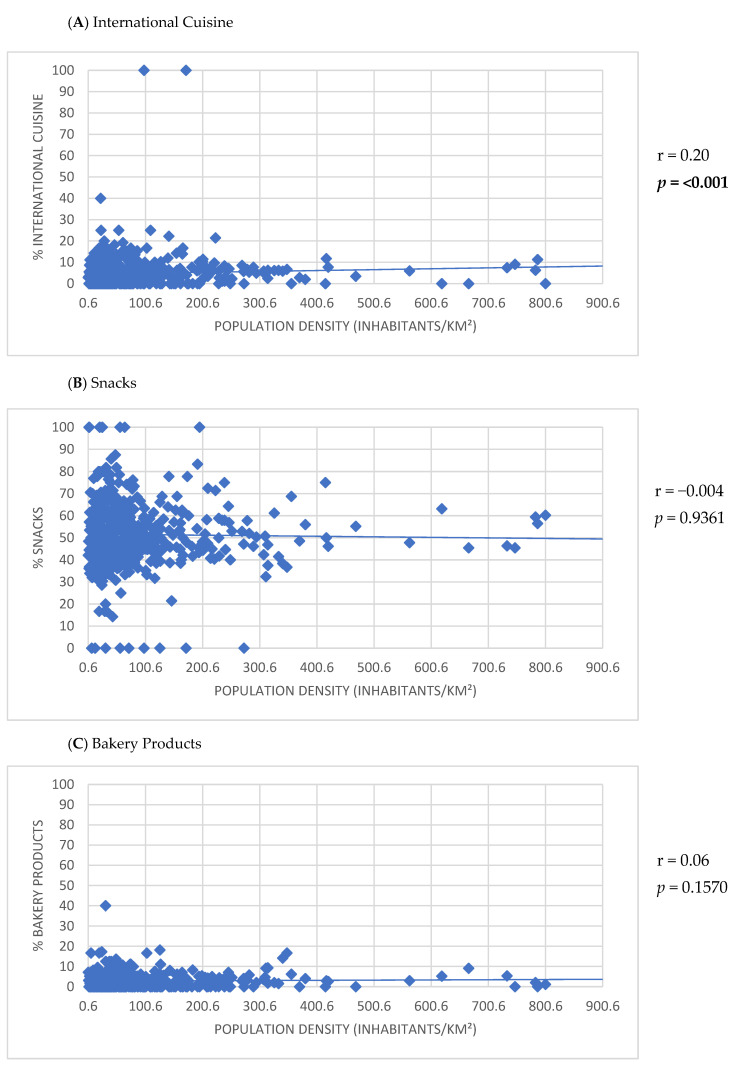

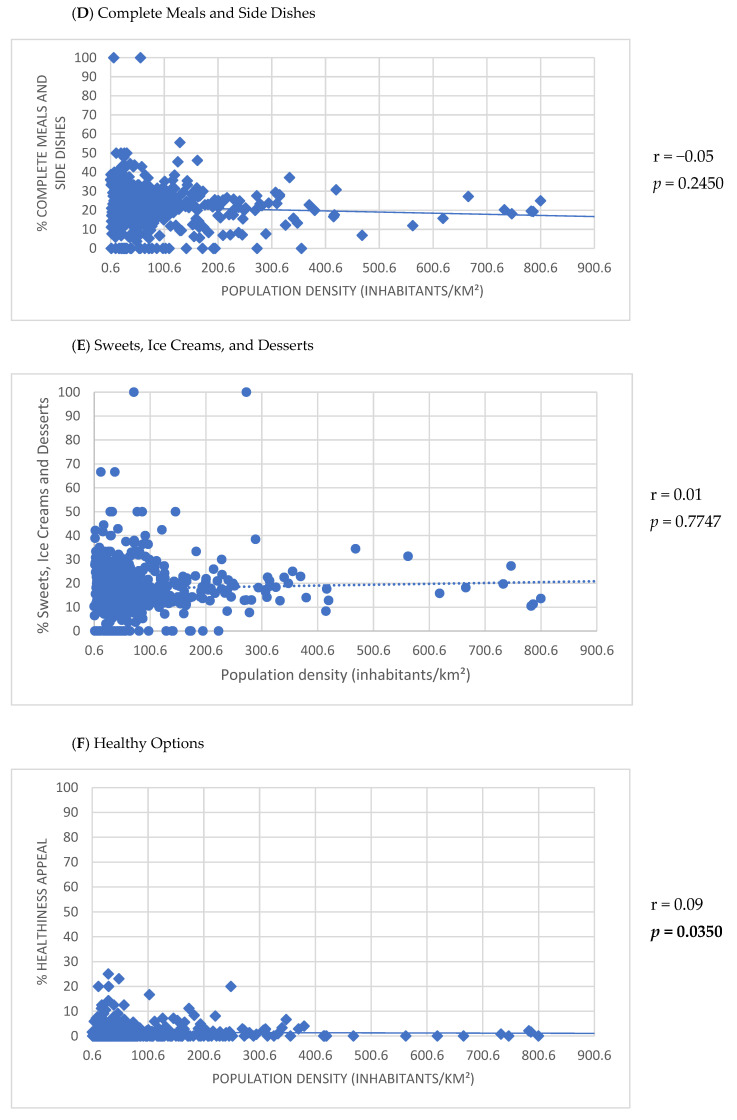
Correlation between the participation of keyword categories for establishments listed in the meal delivery application and the population density of small and medium-sized cities.

**Figure 5:**
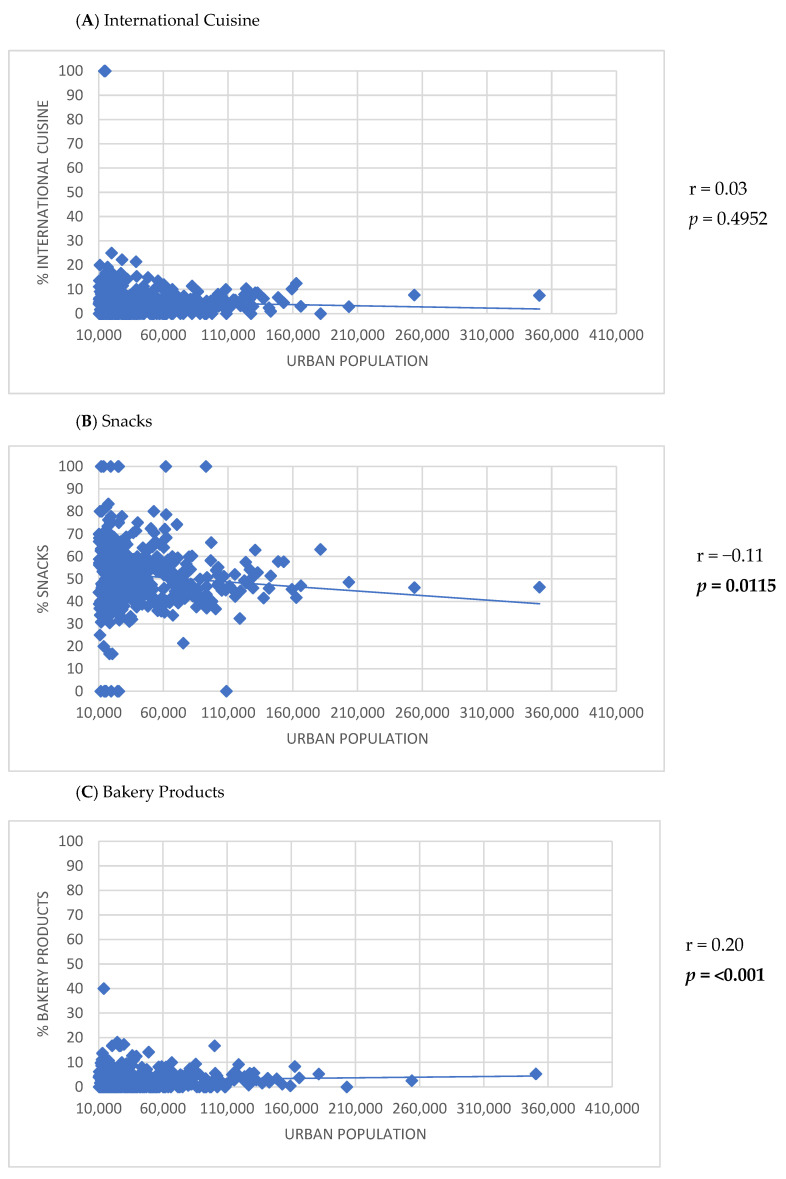

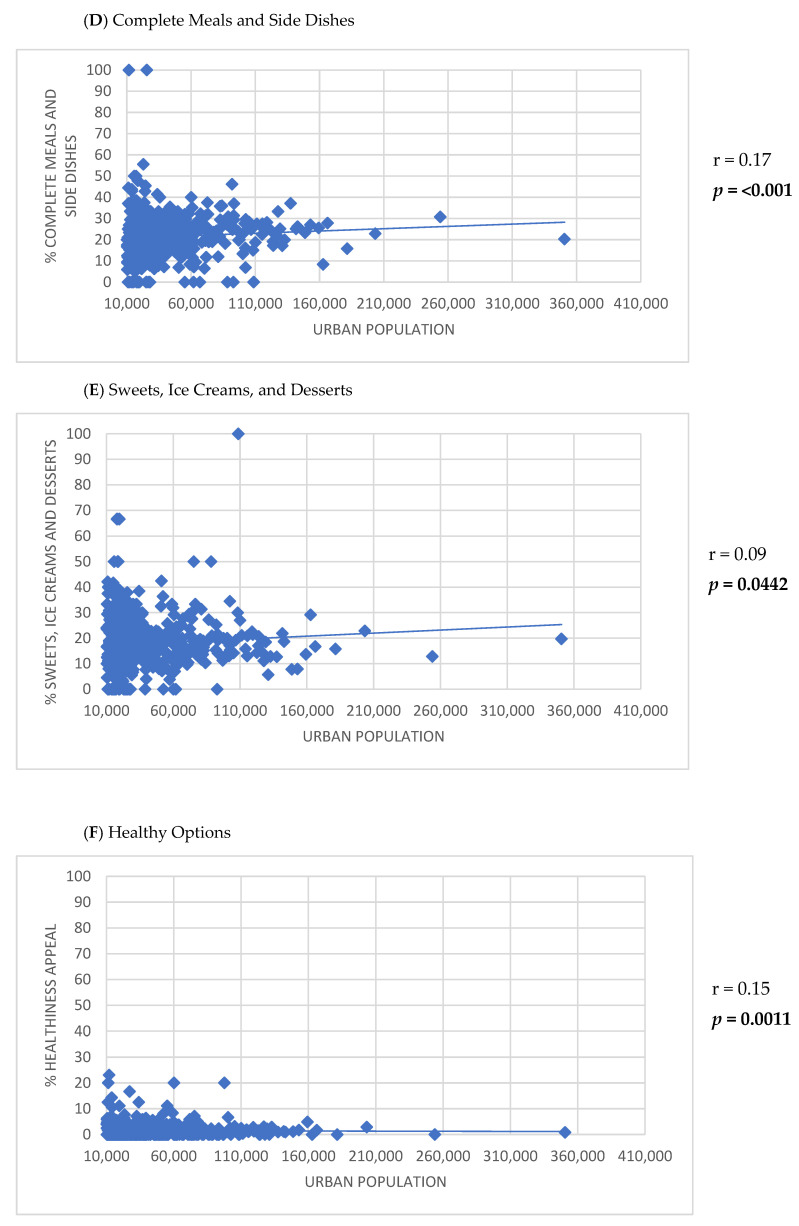
Correlation between the participation of keyword categories for establishments listed in the meal delivery application and the urban population of the studied small and medium-sized cities.

Positive correlations were observed between the number of establishments listed on the app and the HDI-M (r = 0.30; *p* < 0.001), population density (r = 0.23; *p* < 0.001), and urban population size (r = 0.55; *p* < 0.001) (Figure 2). Furthermore, the HDI-M showed positive correlations with the frequency of establishments using the keywords ‘International cuisine’ (r = 0.17; *p* < 0.001) and ‘Healthy options’ (r = 0.17; *p* < 0.001) (Figure 3). Similarly, population density was positively associated with the use of the keywords ‘International cuisine’ (r = 0.20; *p* < 0.001) and ‘Healthy options’ (r = 0.09; *p* = 0.0350) (Figure 4). In contrast, an inverse correlation was found between urban population size and the frequency of use of the ‘Snacks’ category (r = −0.11; *p* = 0.0115). However, urban population size was positively correlated with the use of keywords such as ‘Bakery products’ (r = 0.20; *p* < 0.001), ‘Complete meals and side dishes’ (r = 0.17; *p* < 0.001), ‘Sweets, ice creams, and desserts’ (r = 0.09; *p* = 0.0442), and ‘Healthy options’ (r = 0.15; *p* = 0.0011) (Figure 5).

## 4. Discussion

This study provides a pioneering analysis of the coverage and characteristics of services offered by 28,325 establishments registered on an MDA, encompassing 497 small and medium-sized cities in Brazil. These findings are contextualized within demographic and socioeconomic indicators. Overall, the app’s coverage was more extensive in the South and Southeast regions, whereas the lowest coverage was observed in the North and Northeast regions. Across all Brazilian regions, the ‘Snacks’ keyword category was the most frequently used by establishments, while terms associated with ‘Healthy options’ were the least common.

The predominance of establishments categorized by keywords reflecting unhealthy food options is consistent with previous studies conducted in major cities across developed and developing countries [4,5]. For instance, in cities such as Amsterdam, Chicago, and Melbourne, apps predominantly advertised establishments with keywords such as ‘hamburger’ and ‘pizza’, while healthier options were less common [4]. Similarly, in nine Latin American cities, ‘Snacks’ keywords were among the most frequent, ranging from 15.3% in Mexico City to 45.0% in Montevideo, whereas ‘Healthy’ keywords were used by only 2.4% of establishments in Quito and 8.8% in Montevideo and São Paulo [5]. This global trend of low health-oriented keyword usage highlights the predominance of unhealthy food options on app platforms [9,10,11,12,13,14,15,16,17,18,19,31]. Our findings make a novel contribution to the literature by demonstrating that even in Brazil’s small and medium-sized cities, unhealthy establishments dominate the app’s offerings.

The use of other keyword categories varies regionally. The ‘Complete meals and side dishes’ category was most prevalent in the North and least used in the Southeast, while the ‘Bakery products’ category was more common in the North. In contrast, the ‘Sweets, ice creams, and desserts’ category was frequently used in the Southeast and Midwest. This variation may reflect the strong influence of regional food cultures and dietary habits, particularly in northern Brazil. According to data from the Family Budget Survey (Pesquisa de Orçamentos Familiares—POF) 2017/2018, the North and Northeast regions have lower proportions of ultra-processed foods in household consumption (11.4% and 14.4%, respectively) compared to the national average of 18.5% [32]. Individuals from these regions also have a higher consumption of fresh and minimally processed foods (58.2% in the North and 54.5% in the Northeast) than in the South and Southeast (47.3% and 44.9%, respectively) [32]. The greater availability of establishments offering lower-quality nutritional options via the app in the South and Southeast may reinforce these regional dietary patterns, potentially exacerbating health disparities across populations relying on these services.

Demographic and socioeconomic characteristics of the municipalities were also associated with some characteristics of MDA service. Higher numbers of establishments were registered in municipalities with greater population density and urban populations, consistent with findings from studies in developed countries and large cities. For instance, in Ontario, Canada, a positive relationship was found between the total number of app-listed retailers and city population size and density [12]. Similarly, in Latin America, the presence of establishments on an app was positively associated with the number of inhabitants [5]. Regarding the relationship between the number of establishments and HDI-M, the literature reports mixed findings. In Amsterdam and Melbourne [4], as well as in cities across seven European countries [21], higher availability of online food delivery services was observed in high-income areas. However, in England, studies found no significant differences in the availability of online food outlets between low- and high-income neighborhoods [21]. A study involving the app Just Eat in England reported greater availability in less affluent areas [22]. In Brazil, research conducted in a state capital revealed higher coverage of app-listed establishments in areas with greater social vulnerability [31]. These findings underscore the need for further research to better understand how apps allocate services across areas with varying income levels and to monitor these dynamics, given the adaptability of digital platforms to market demands.

In the context of small and medium-sized cities, variations in app coverage may be attributed to several factors. The Southeast and South regions had the highest app coverage, partly due to their demographic and economic characteristics. For example, the Southeast contains São Paulo and Minas Gerais, two of Brazil’s most populous states [33], and is home to the largest number of app users in 2020 [34]. Paraná, located in the South, is one of the country’s largest states, with 11,443,208 inhabitants [33], and hosts the app’s headquarters, facilitating service management and organization [26]. Additionally, urban areas with higher population densities tend to have greater demand for convenient meal options offered by MDAs, driven by time constraints on meal preparation [35,36]. Internet access is another critical factor influencing app coverage. While 77% of urban areas in Brazil have internet access, this figure drops to 53% in rural areas. Furthermore, internet access rates are lowest in the North (71%) and Northeast (74%) regions compared to the Midwest, South, and Southeast (75–76%) [37]. Transportation logistics and infrastructure also likely contribute to the observed disparities, as these factors are more developed in the Southeast and South compared to the North, Northeast, and parts of the Midwest [38].

The use of certain keyword categories, such as ‘International cuisine’ and ‘Healthy options’, was also different across cities, being higher in regions with greater HDI-M and population density. Also, positive correlations were observed between urban population and the use of keywords such as ‘Bakery products’, ‘Complete meals and side dishes’, ‘Sweets, ice creams, and desserts’, and ‘Healthy options’, while a negative correlation was found for ‘Snacks.’ For instance, in São Paulo, the biggest city in the country, fast-food establishments, food trucks, pastry shops, and pizzerias were more prevalent in neighborhoods covered by MDAs with lower HDI-M, whereas restaurants were concentrated in higher HDI-M areas [39]. These findings highlight inequalities in the types of establishments accessible via apps and suggest disparities in access to different types of establishments.

Finally, we discuss the strengths and limitations of this study. It included all small and medium-sized cities served by the app, encompassing all registered establishments, thereby providing a comprehensive understanding of the app’s coverage among small and medium-sized cities in Brazil. It also explored how socioeconomic and demographic factors are associated with an MDA service in these cities. However, some limitations must be acknowledged. The analysis was limited to a single app, potentially underestimating the coverage provided by other platforms, including proprietary restaurant apps. Additionally, the reliance on keywords to characterize establishments may not fully capture the diversity of items on their menus.

## 5. Conclusions

The study demonstrates for the first time that small and medium-sized cities in Brazil are covered by food delivery app services. In the app studied, greater coverage of establishments was noted in more populous cities and higher HDI-M levels. Moreover, the majority of establishments predominantly identified with keyword categories associated with unhealthy eating across all cities and regions covered by the app. However, variations in other keyword categories were noted, reflecting differences in the demographic and socioeconomic characteristics of the municipalities.

The methodology employed in this study can be applied in future research seeking to collect large-scale data on MDA coverage and the characteristics of food establishments. Additionally, future studies should explore the relationship between keywords and actual menu offerings to improve food classification accuracy and streamline analytical processes. Further investigations into the most frequently ordered items by consumers in small and medium-sized cities using this app could provide valuable insights into how the platform shapes food consumption patterns.

In terms of public policy development, this study highlights the need for measures to reduce inequalities in MDA service coverage across locations with varying demographic and socioeconomic profiles. Specifically, it underscores the importance of ensuring equitable access to healthy food options, thereby contributing to a more inclusive and health-promoting digital food environment.

## Figures and Tables

**Table 1:** Categorization of keywords assigned to establishments in the meal delivery application.

Keyword Categories	Keywords
International cuisine	Arab–Asian–Chinese–Combined–Fondue–Hawaiian–Italian–Japanese–Kebab–Mediterranean–Mexican–Peruvian–Sashimi–Shawarma/Kebab–Sushi–Tacos–Thai–Temaki–Yakimeshi–Yakisoba
Snacks	Bauru–Beirut–Calzone–Coxinhas–Crepe–Empadas–Empanadas–Esfiha–Hamburger–Hot Dog–Kachurrasco–Kibbeh–Snack–Pastry–Pizza–Cone Pizza–Savory snacks–Sandwich–Xis–Wrap
Bakery products	Cake–Croissant–Crepioca–Cuscuz–Bakery–Bread–Pancake–Pamonhas–Cheese bread–Quitanda–Tapioca–Waffle
Complete meals and side dishes	Acarajé–À La Minute–Side Dish–Rice–Roasted–Codfish–French Fries–Stuffed Potato–Swiss Potato–Broth–Shrimp–Meats–Barbecue–Contemporary–Brazilian Cuisine–Starter–Entrevero–Casserole–Skewers–Feijoada–Filet–Chicken–Roasted Chicken–Fried Chicken–Seafood–Galeto–Grilled–Lasagna–Macaroni–Meal box–Omelette–Parmegiana–Pasta–Fish–Appetizer–Portions–Complete meal–Dishes–Special Dishes–Executive Dishes–Cheese–Quentinhas–Meal–Risottos–Soups–Stroganoff
Sweets, ice creams, and desserts	Açaí–Churros–Cookies–Sweets–Donuts–Frozen–Milkshake–Paletas–Popsicles–Desserts–Ice Cream–Pie
Healthy options	Fit and Healthy–Light–Salad–Fruit Salad–Healthy–Gluten-Free–Juice–Vegan–Vegetarian–Smoothie

Note: Açaí is a food deeply rooted in Brazilian culinary culture and is widely consumed in the Northern region of the country. However, we chose to allocate the keyword in the category “Sweets, ice creams, and desserts” since, in most regions of the country, it is consumed with added sugar (guaraná syrup) and/or accompanied by other ultra-processed treats.

**Table 2:** Meal delivery application coverage in small and medium-sized cities across Brazilian regions.

Region	Cities Served by the App	Small and Medium-Sized Cities	% of Small and Medium-Sized Cities Served by the App ^a^
Midwest	58	462	12.55
Northeast	41	1783	2.30
North	11	447	2.46
Southeast	225	1651	13.63
South	162	1184	13.68
Total	497	5527	8.99

App: meal delivery application. ^a^ (Number of small and medium-sized cities in the region served by the app/total number of small and medium-sized cities in the region) × 100.

**Table 3:** Distribution of keyword categories for establishments listed in the meal delivery application across Brazilian regions.

Region/State	Keyword—Participation (%) Among Establishments					
	International Cuisine	Snacks	Bakery Products	Complete Meals and Side Dishes	Sweets, Ice Creams, and Desserts	Health-Oriented Options
Midwest	3.61	44.07	4.15	27.85	19.64	0.68
Northeast	3.96	48.18	4.41	26.15	15.91	1.39
North	3.34	45.26	5.34	32.84	11.48	1.74
Southeast	4.62	48.26	3.33	23.03	19.24	1.52
South	5.23	51.88	2.62	24.11	14.60	1.57

## Data Availability

Data will be made available upon request.

## Supplementary Materials

The following supporting information can be downloaded at: https://www.mdpi.com/article/10.3390/ijerph22020293/s1. Supplementary Material S1: Frequency of unique keywords within each category.

## Abbreviation

MDA: Meal delivery application
