# Supplementary material for: Coverage and Characterization of Food Delivery Services Through a Mobile Application in Small and Medium-Sized Cities in Brazil
Source: Int J Environ Res Public Health. 2025 Feb 16;22(2):293. doi: 10.3390/ijerph22020293 (PMC11855524; doi:10.3390/ijerph22020293)
Supplement: Supplementary file 1 [file ijerph-22-00293-s001.zip › ijerph-3442887-supplementary.pdf]

Supplementary Material S1: Frequency of unique keywords, within each category.

Keyword Categories	n	%
**International Cuisine**		
Arab	73	0.26
Asian	14	0.05
Chinese	44	0.16
Combined	7	0.02
Fondue	11	0.04
Hawaiian	7	0.02
Italian	30	0.11
Japanese	548	1.93
Kebab	4	0.01
Mediterranean	1	0.00
Mexican	65	0.23
Peruvian	1	0.00
Sashimi	13	0.05
Shawarma/Kebab	62	0.22
Sushi	311	1.10
Tacos	6	0.02
Thai	1	0.00
Temaki	24	0.08
Yakimeshi	1	0.00
Yakisoba	75	0.26
**Snacks**		
Bauru	19	0.07
Beirut	15	0.05
Hot dog	225	0.79
Calzone	43	0.15
Coxinhas	82	0.29
Crepe	109	0.38
Empadas	56	0.20
Empanadas	6	0.02
Esfiha	342	1.21
Hamburger	3,977	14.04
Hot Dog	438	1.55
Kachurrasco	2	0.01
Kibbeh	5	0.02
Snack	3,338	11.78
Pastry	758	2.68
Pizza	3,021	10.67
Cone Pizza	38	0.13
Savory snacks	834	2.94
Sandwich	319	1.13
Xis	172	0.61
Wrap	3	0.01
**Bakery Products**		

Cake	388	1.37
Croissant	14	0.05
Crepioca	3	0.01
Cuscuz	38	0.13
Bakery	178	0.63
Bread	38	0.13
Pancake	75	0.26
Pamonhas	27	0.10
Cheese bread	74	0.26
Quitanda	9	0.03
Tapioca	93	0.33
Waffle	17	0.06
**Complete Meals and Side Dishes**		
Acarajé	22	0.08
À La Minute	48	0.17
Side Dish	13	0.05
Rice	11	0.04
Roasted	63	0.22
Codfish	2	0.01
French Fries	177	0.62
Stuffed Potato	257	0.91
Swiss Potato	16	0.06
Broth	123	0.43
Shrimp	20	0.07
Meats	259	0.91
Barbecue	215	0.76
Contemporary	4	0.01
Brazilian Cuisine	118	0.42
Starter	4	0.01
Entrevero	1	0.00
Casserole	21	0.07
Skewers	482	1.70
Feijoada	47	0.17
Filet	8	0.03
Chicken	80	0.28
Roasted Chicken	90	0.32
Fried Chicken	163	0.58
Seafood	12	0.04
Galeto	9	0.03
Grilled	11	0.04
Lasagna	49	0.17
Macaroni	123	0.43
Meal box	2,462	8.69
Pasta	408	1.44
Omelette	10	0.04
Parmegiana	33	0.12
Pasta	3	0.01
Fish	101	0.36

Appetizer	88	0.31
Portions	700	2.47
Complete meal	37	0.13
Dishes	79	0.28
Special Dishes	47	0.17
Executive Dishes	70	0.25
Cheese	25	0.09
Quentinhas	87	0.31
Meal	268	0.95
Risottos	29	0.10
Soups	20	0.07
Stroganoff	19	0.07
**Sweets, Ice Creams and Desserts**		
Açaí	2,476	8.74
Churros	135	0.48
Cookies	56	0.20
Sweets	1,235	4.36
Donuts	63	0.22
Frozen	2	0.01
Milkshake	75	0.26
Paletas	9	0.03
Popsicles	44	0.16
Desserts	160	0.56
Ice Cream	646	2.28
Pie	33	0.12
**Healthiness Appeal**		
Fit and Healthy	228	0.80
Light	1	0.00
Salad	37	0.13
Fruit Salad	17	0.06
Healthy	33	0.12
Gluten-Free	5	0.02
Juice	42	0.15
Vegan	22	0.08
Vegetarian	15	0.05
Smoothie	3	0.01
